# Vesicles in Multiple Shapes: Fine-Tuning Polymersomes’ Shape and Stability by Setting Membrane Hydrophobicity

**DOI:** 10.3390/polym9100483

**Published:** 2017-10-02

**Authors:** Jens Gaitzsch, Lea Messager, Eloise Morecroft, Wolfgang Meier

**Affiliations:** 1 Departement of Chemistry, University of Basel, Mattenstrasse 24a, BPR1096, 4058 Basel, Switzerland; 2 LAGEP-Université Claude Bernard Lyon 1, 43 Boulevard du 11 novembre 1918, Bâtiment CPE-308G, M69622 Villeurbanne Cedex, France; lea.messager@univ-lyon1.fr; 3 Institute of Chemical Biology, Exhibition Road, London SW7 2AZ, UK; eloise.morecroft.10@alumni.ucl.ac.uk

**Keywords:** self-assembly, polymersomes, temperature-sensitivity, ATRP

## Abstract

Amphiphilic block-copolymers are known to self-assemble into micelles and vesicles. In this paper, we discuss the multiple options between and beyond these boundaries using amphiphilic AB diblock and ABC triblock copolymers. We adjust the final structure reached by the composition of the mixture, by the preparation temperature, and by varying the time-scale of formation. This leads to the formation of vesicles and micelles, but also internal micelles in larger sheets, lamellar vesicles, and closed tubes, thus broadening the amount of self-assembly structures available and deepening our understanding of them.

## 1. Introduction

Polymersomes have shown great potential over the last two decades in the fields of biomimetic self-assembly, drug delivery, and energy. This is because polymersomes show interesting properties in comparison with their lipid counterparts. They are known to be chemically and mechanically more stable than liposomes [1,2,3,4,5,6]. Polymer vesicles can also withstand mechanical forces [7,8] and have a membrane that can not only withstand greater pressure than liposomes but have also proven to be of low fluidity [9,10,11]. All of these facts are in line with their improved stability over liposomes. The same features are observed for polymeric micelles in comparison with their lipid counterparts. This is not surprising as both gain their stability from the fact that the polymer chains entangle with one another, and the assemblies are thus non-ergodic, making it harder to break them apart [5,12,13]. The ability of amphiphilic block-copolymer to form vesicles or micelles is mainly governed by the block-length ratio of the hydrophilic block to the hydrophobic one. The longer the hydrophobic block becomes, the more likely vesicles are [6,14,15]. This has been proven by comparing polymers of the same composition of the blocks but with different block-length ratios. Using polymerisation-induced self-assembly (PISA), the various self-assembly states of an amphiphilic block-copolymer are studied during its polymerization [16,17,18,19]. Such studies as well as studies on vesicle evolvement from a bulk polymer always show a transition from micelles (also multi-compartment micelles) to polymersomes via worms and jellyfish-like structures [14,20,21]. Once vesicles are formed, a vastly longer hydrophobic block either leads to larger vesicle walls or the formation of genus particles as intermediates but ultimately to the precipitation of the material [22,23]. All these transitions have been reached by comparing amphiphilic block-copolymers of the same chemical structure of the repeating units but different block length ratios. Only limited studies are available whether the transition mentioned can also be achieved by mixing block-copolymers of different compositions to fine-tune the hydrophobicity and thus influence the hydrophilic-to-hydrophobic balance. It is likely that such a change would also induce the formation of a certain self-assembled structure or cause it to change from one to another, maybe even causing the formation of new self-assembled structures. Some examples in literature have actually shown that this mixing can lead to a whole new class of vesicles, such as raspberry-shaped ones or polymersomes with multiple surface domains [24,25,26,27]. Both effects were the result of phase separation on the vesicle surface. In this work, we wanted to avoid phase separation, but induce the formation of different homogeneous self-assembled structures by slight changes of the hydrophobic part, potentially proving that there is a fine balance that determines vesicle stability or the formation of more complex self-assembled structures.

## 2. Experimental Section

The polymers, poly(ethylene glycol)-*block*-poly(hydroxylpropyl methacrylate) (PEG_45_-PHPMA_100_), poly(ethylene glycol)-*block*-poly(hydroxylpropyl methacrylate)-*block*-poly(diisopropylaminoethyl methacrylate) (PEG_45_-PHPMA_70_-PDPA_30_), and poly(methacrylic phosphoryl choline)-*block*-poly(hydroxylpropyl methacrylate)-*block*-poly(diisopropyl aminoethyl methacrylate) PMPC_25_-PHPMA_55_-PDPA_8_ were prepared using atom transfer radical polymerisation (ATRP) according to a procedure described previously [25]. Synthesis of the initiators PEG-Br [28] for the PEG-based polymers and ME-Br [29] for the PMPC-based polymer was also adopted from literature. A detailed description can be found in the SI alongside how compositions were estimated from NMR. (More details available in the Supporting Information (SI) – 2.1 for synthesis, 2.2 for NMR, 2.3 for calculations from NMR, Figure S1 for GPC traces)

Self-assembly was performed by dissolving 10 mg of the corresponding polymer or polymer mixtures in a chloroform/methanol (3:1) mixture. The solvent was then evaporated and film rehydrated in 2 mL of water by stirring the solution for the time points at the temperatures stated in the manuscript before TEM imaging. For more details on Materials and Methods, please see SI, Sections 1.1 and 1.2.

## 3. Results and Discussion

**Figure 1:**
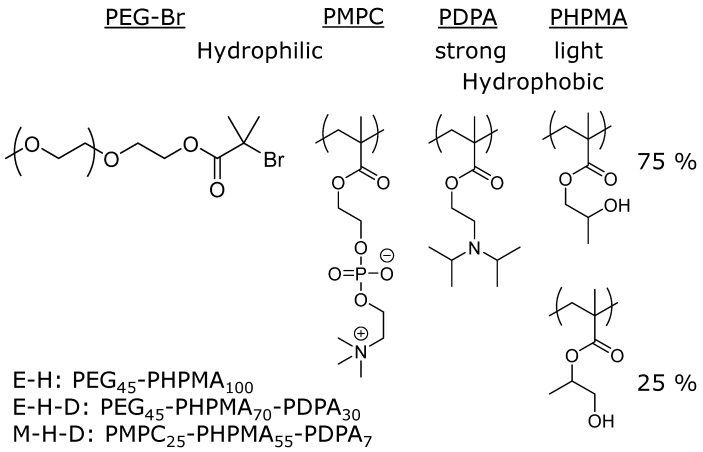
Chemical structures of the polymer blocks used in our studies. PEG and PMPC are hydrophilic blocks, while PHPMA and PDPA are hydrophobic blocks with PDPA being the stronger hydrophobic one.

We used three different amphiphilic AB and ABC block-copolymers and investigated how they self-assemble alone or when mixed together. The first block-copolymer was an AB diblock, namely PEG_45_-PHPMA_100_ (Figure 1, E-H). This polymer had a similar block-length ratio as ones reported by Armes et al., who reported that this polymer forms vesicles using PISA. We also synthesized two ABC triblock-copolymers based on PEG-PHPMA, which could significantly impact the vesicle formation process and lead to different self-assembled structures. The first polymer for this purpose was PEG_45_-PHPMA_70_-PDPA_30_ (Figure 1, E-H-D). This polymer contains a block of PDPA that is more hydrophobic than PHPMA. In contrast to HPMA, DPA monomers are already insoluble in water, indicating that PDPA is more hydrophobic than PHPMA. We wanted to make sure that only the change in hydrophobicity had an influence and thus kept the total number of repeating units within the hydrophobic part similar to the original polymer. As this would only disrupt the hydrophobic part, we also used PMPC_25_-PHPMPA_53_-PDPA_7_ (Figure 1, M-H-D), replacing the PEG with PMPC. It is known that PEG and PMPC are not miscible and phase-separate rapidly on the surface of polymersomes, leading to polymersomes with an inhomogeneous surface [9]. We wanted to enhance this effect by using a micelle-forming polymer with PMPC. In order to be micelle-forming, the hydrophobic part of this block-copolymer was shortened in comparison to E-H. The three polymers were subsequently used to study their self-assembly behaviour with different mixtures at different temperatures. We will use different temperatures because PHMPA is temperature-sensitive as the hydrogen bonds originating from the hydroxyl unit become weaker with elevated temperatures [16,30,31]. PDPA is also temperature-sensitive as its p*K*a value varies with temperature, making it completely hydrophilic at 5 °C [23]. Most amine units are then protonated at a neutral pH. We analyze our copolymer mixtures at room temperature (approx. 23 °C) and at 50 °C. Battaglia et al. demonstrated that PDPA is already a suitable hydrophobic block for self-assembly at the first temperature [32,33,34]. The same group also reported that raising the temperature affected the self-assembly of PMPC-PDPA diblock-copolymers [23]. Heating the sample to 50 °C could thus also influence the self-assembly process in our case.

**Figure 2:**
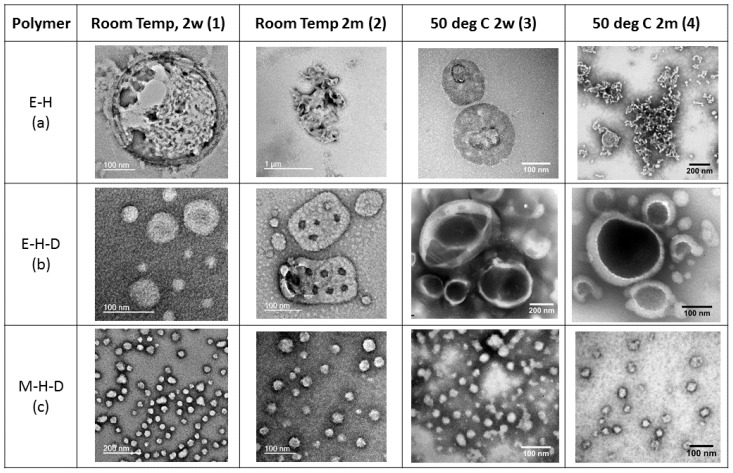
TEM images of the self-assembly structures of our block-copolymers: PEG-PHPMA (E-H, (**a**)), PEG-PHPMA-PDPA (E-H-D, (**b**)), and PMPC-PHPMA-PDPA (M-H-D, (**c**)). Images were taken at different conditions, namely at room temperature after 2 weeks (**1**) and 2 months (**2**) as well as at 50 °C after 2 weeks (**3**) and 2 months (**4**).

We conducted all of our self-assembly experiments starting from a thin film, which was then hydrated under vigorous stirring. This technique well established and the self-assembly process is generally well studied (please see section 2.5 with Figure S9 in the SI for details) [15,35]. Our starting point was the self-assembly of the single polymers to determine their structural organization. Whereas PEG-PHPMA self-assembly is already studied in detail using PISA [17], it has not yet been studied thoroughly by a top-down approach. We observed vesicles with an additional membrane layer, or multiple layers of a membrane curled up inside of the vesicle (Figure 2(a1)). Vesicles of PEG-PHPMA with a multi-layered wall have been reported in the PISA approach, but only for high concentrations (20 wt %). We observed similar structures at 5 mg/mL, so 0.5 wt %. Similar to PISA [17], these structures are not stable and decay over time to curled-up membrane fragments (Figure 2(a2)). This shows that PHPMA is not hydrophobic enough to support a stable polymersome membrane. This effect could be explained by the hydroxyl group present in each repeating unit of PHPMA, which can form hydrogen bonds with water, affording it some hydrophilicity. If self-assembled at 50 °C, vesicular structures are formed by this polymer (Figure 2(a3)). Raising the temperature is known to break hydrogen bonds. Hence, a self-assembly process at elevated temperatures increases the hydrophobicity of PHMPA, pushing the polymer towards vesicle formation. However, stirring at 50 °C for two months leads to disintegration of the vesicle, leaving curled-up membranes, as was already observed at room temperature (Figure 2(a4)–DLS in Figure S5 of the SI). The samples here were prepared on a cold grid, so the samples were instantaneously cooled from 50 °C to room temperature, which might have had an impact on their final structure. However, since vesicles were observed for the sample prepared at 50 °C after a few days, this effect seems less significant than the actual stability of the PEG-PHPMA self-assemblies over time.

We conclude that vesicles of PEG-PHPMA are only stable for a limited period of time and only at elevated temperatures.

The self-assembly of PEG-PHPMA-PDPA (E-H-D) triblock was then investigated. Thirty units of PHPMA were replaced by PDPA in comparison with the E-H diblock copolymer. As PDPA is more hydrophobic, we postulated that the resulting vesicles would be more stable. We indeed observed nanoparticles in the size-range of vesicles or multicompartment micelles [21] for the PEG-PHPMA-PDPA triblock-copolymer after two weeks of film hydration at room temperature (Figure 2(b1)–DLS in Figure S3 of the SI). They are also unstable, but evolve into nanoparticles with internal holey structures after two months. We suggest that these holes are reverse micelles within the self-assembly structures (here named “waffle-vesicles”) (Figure 2(b2)–DLS in Figure S4 of the SI). Our claim is based on observations in earlier studies, where Battaglia et al. reported similar structures for polymers with increasing hydrophobic block length [23]. In that case, the formation of internal concave shapes was attributed to the great difference between the hydrophilic and hydrophobic chain segments, which destabilizes the membrane curvature, creating internal defects or holes. Looking at the geometry of common self-assembly structures, vesicles are the least convex spherical structures, followed by tubes (no curvature in one dimension), which are then followed by concave shapes such as reverse micelles. As a consequence, the internal micelles in the waffle vesicles in Figure 2(b2) could represent reverse micelles within previously formed (and merged) vesicles. In our case, replacing PHPMA with PDPA did not only increase the hydrophobicity, but also the molecular weight of the final polymer. We thus suggest that the hydrophilic to hydrophobic balance just above the threshold of stable vesicles (for E-H-D in comparison to E-H) explaining the formation of internal micelles and the resulting waffle like structure. Another effect is the phase separation of PDPA and PHPMA due to their difference in hydrophobicity, similar to the known phase separation of PEG and PMPC on the corona of vesicles [9,25]. Both the hydrophilic-to-hydrophobic mismatch and the phase separation aid the formation of the reverse micelles observed.

A self-assembly at 50 °C would decrease the difference in hydrophobicity since heating the sample makes the PHPMA more hydrophobic. A difference in hydrophobicity could still remain in the end, because PDPA is less protonated and thus also more hydrophobic at 50 °C [23]. However, heat also promotes chain movement due to the increased thermal energy. Phase separation could thus be faster, but with an increased hydrophobicity for both PHPMA and PDPA. We then expected to see genus-like waffle particles, as reported before [23]. Instead, we observed giant donut-like structures that were stable over time (Figure 2(b3,b4))–DLS of b4 in Figure S7 of the SI). The internal core of these donuts attracts a majority of the staining agent added to the solution (PTA on our case). PTA preferentially stains ester groups, so PDPA or PHPMA in our case. This leads to the conclusion that the internal micelles of the waffle vesicles observed for prolonged stirring at room temperature expanded to produce a single hole. Heating makes the PHPMA part more hydrophobic and the chains more mobile, allowing the mentioned expansion to occur. The additional hydrophobicity of the system prevents the formation of normal vesicles.

The effect of the replacement of PEG block with PMPC on the triblock copolymer was then studied on PMPC-PHPMA-PDPA (M-H-D). This polymer formed micelles throughout the entire series, even upon heating and prolonged times (Figure 2(c1–c4)). This was expected as switching to a more hydrophilic block would destabilise the vesicles and induce a switch to micelles or worms. Interesting enough, we observed no influence of phase separation between PHMPA and PDPA for this system. We concluded that this effect is only important when vesicular structures are formed but micelles are stable. It did, however, appear to us that the micelles became less stable over time for higher temperatures as the contrast became less pronounced, but did not study this in further detail.

As an intermediate conclusion, the self-assembly of PEG-PHPMA and PEG-PHPMA-PDPA, or of PMPC-PHPMA-PDPA alone, resulted in unstable vesicles or micelles. We postulated that a possible stabilisation of the PEG-PHPMA assemblies with small amounts of one of our triblock-copolymers would establish a stable phase. We thus added either 5 wt % PEG-PHPMA-PDPA or 5 wt % PMPC-PHPMA-PDPA to the plain PEG-PHPMA in order to favour the self-assembly towards micelles or vesicles. However, due to the complex self-assembly behaviour of pure PEG-PHPMA-PDPA just described, similar or other complex self-assemblies could not be excluded.

**Figure 3:**
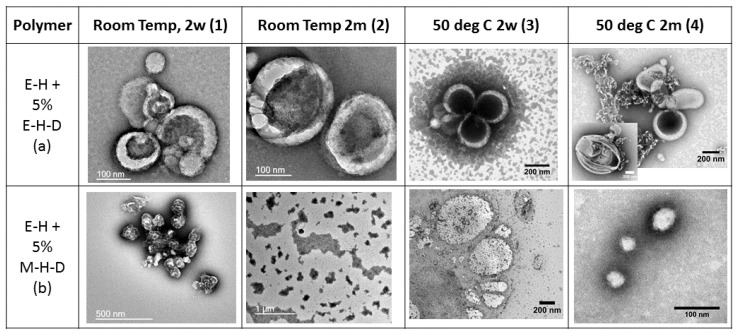
TEM images of the mixture of block-copolymers. PEG-PHPMA + PEG-PHPMA-PDPA (**a**) and PEG-PHPMA + PMPC-PHPMA-PDPA (**b**) at room temperature for 2 weeks (**1**) and for 2 months (**2**), and at 50 °C for the same time periods (**3** and **4**, respectively).

Adding PEG-PHPMA-PDPA was to stabilise vesicles for two reasons. First of all, the higher hydrophobicity of PDPA would push the self-assemblies towards vesicles. Secondly, the self-assembly of the pure triblock copolymer gave vesicles at room temperature. However, Figure 3(a1) (DLS in Figure S6 of the SI) shows that the self-assembly of this mixture at room temperature yielded derivatives of the donut-like structures, which were already present for the pure PEG-PHPMA-PDPA at 50 °C (Figure 2(b3)). While the pure triblock self-assembled into circles in that case, the mixture shows self-assembly into a half-moon-shape rather than a complete circle. This rather unexpected behaviour can only be the consequence of a small amount of PDPA added to the system. This small addition drives the spontaneous organization towards genus-like particles. Apparently, starting phase separation of PHPMA and PDPA already disturbs the geometry necessary for vesicles to be formed. Within this theory, the phase separation should continue and slowly close the half-moon structures over time to produce closed genus-like particles. The observation of the same sample after two months indicates that this hypothesis is confirmed. The outer shell of the genus particles is visible, as is the inner core of it, supporting our theory (Figure 3(a2)).

Since the phase separation already gave genus-like particles at room temperature, self-assembly at a higher temperature ought to show a similar trend. As discussed above, accelerated phase separation resulting from higher chain mobility was likely to occur. Meeting this expectation, we made a similar observation for the PEG-PHPMA/PEG-PHPMA-PDPA mixture at 50 °C as compared to a self-assembly at room temperature. Interestingly, the observed half-moon structures are similar to the ones observed at room temperature (Figure 3(a3)). Hence, for the first time, we did not see a distinct temperature dependency with the PEG-PHPMA-based self-assembly. However, if left for two months, a partial decline in stability is observed, showing that the self-assembly mixture is more unstable at 50 °C than at room temperature. This is in line with the previously indicated increased mobility of the polymer chains, which decreases the stability of all PEG-PHPMA self-assemblies at an elevated temperature. The half-moon structures are present, but so are decomposed vesicles (Figure 3(a4)–DLS in Figure S8 of the SI). Additionally, some multi-walled vesicles are present, which visibly decompose further into small aggregates, the products of the disintegration process. This leads to the conclusion that such multi-walled vesicles are intermediate self-assembly structures between stable vesicles and decomposed smaller aggregates. As already observed with pure PEG-PHPMA, these self-assemblies are not stable themselves, but disassemble into smaller aggregates over time.

PEG-PHPMA-PDPA already had a deciding influence on the self-assembly, even though we added only 5 wt % of the polymer to PEG-PHPMA. Adding PMPC-PHPMA-PDPA would then have to destabilise the self-assembly to produce micelles or instable aggregates. Indeed, we then only saw unstable self-assemblies. We observed decomposed vesicles at room temperature after two weeks (Figure 3(b1)) as well as after two months (Figure 3(b2)) or the vesicles that show signs of decomposition at 50 °C after two weeks of stirring (Figure 3(b3)). The only surprise was the presence of micelles present after two months of stirring at 50 °C (Figure 3(b4)–DLS in Figure S2 of the SI). While the previous vesicles in this study repeatedly decomposed directly into smaller aggregates, this mixture devolves into micelles. It can be concluded that the PMPC-PHPMA-PDPA triblock present in the mixture destabilises larger self-assemblies, even if only 5 wt % concentrations are added to the PEG-PHPMA diblock copoplymer.

## 4. Conclusions

We have shown how different degrees of hydrophobicity in an amphiphilic block-copolymer can significantly influence the resulting shape and stability of the self-assembled structures. While pure PEG-PHPMA forms vesicles at elevated temperatures, these vesicles were as unstable as multi-walled vesicles formed at room temperature. An addition of a more hydrophobic block such as PDPA to form a PEG-PHPMA-PDPA triblock copolymer yielded more complex aggregates. At room temperature, the more hydrophobic PDPA separates out of the vesicles to form internal reverse micelles. At higher temperatures, however, the PHPMA becomes more hydrophobic, stopping the phase separation from PDPA, but forcing the formation of closed and open donut-like self-assemblies. Hence, the addition of PDPA stabilises these particles and generally supports larger self-assembly structures. On the contrary, making the hydrophilic part slightly longer and more hydrophilic (switching from PEG to PMPC) destabilises the self-assembly into vesicles and micelles were obtained over time and within a certain temperature range. The mixture of these copolymers resulted in additional complex aggregates that were mostly due to the contribution of the incorporated triblock. We have thus demonstrated how the shape and stability of self-assembled nanoparticles can be tailored by the block-copolymer composition and mixtures.

## Supplementary Materials

The following are available online at www.mdpi.com/2073-4360/9/10/483/s1.
