# Supplementary material for: Vesicles in Multiple Shapes: Fine-Tuning Polymersomes’ Shape and Stability by Setting Membrane Hydrophobicity
Source: Polymers (Basel). 2017 Oct 2;9(10):483. doi: 10.3390/polym9100483 (PMC6418632; doi:10.3390/polym9100483)
Supplement: Supplementary file 1 [file polymers-09-00483-s001.pdf]

Supporting Information

1. Materials and Methods

1.1. Materials

2-(Methacryloyloxy)ethyl phosphorylcholine monomer (MPC, 99.9% purity) was donated by Biocompatibles U.K. Ltd. Diisopropylaminoethyl methacrylate (DPA), methoxy-poly(ethylene glycole) PEG-OH, Mn = 2000 g/mol), 2-hydroxypropylmethacrylate (HPMA), silica gel 0.2–0.5mm 30-70 mesh chromatograph), triethyl amine, alpha-bromo-isobutyric acid bromide, phosphotungstenic acid (PTA), copper(I) bromide (Cu(I)Br, 99.999%), 2,2′-bipyridine (bpy, 99%) and chloroform were purchased from Sigma-Aldrich UK. Ethanol (Normapur) and methanol (Normapur) were purchased from Merck KG (Darmstadt, Germany). All of the above were used as received.

Phosphate-buffered saline (PBS) was prepared from tablets obtained from Oxoid (Basingstoke, UK). Semi-permeable cellulose dialysis tubing (Spectra/Por 6 MWCO 1,000) was purchased from Spectrum Labs (Breda, Netherlands).

1.2. Methods

Gel permeation chromatography was carried out using a Malvern Viskotek GPC system (Malvern Instruments, UK) using a Novema Max 100Å Column with a Novema Max Guard Column (both PSS Polymer, Germany) with 0.25 vol % TFA in water as an eluent or a Resipore 100Å Column with a Resipore Guard Column (Agilent Technologies, USA) with a chloroform/methanol (3:1) eluent.

NMR spectroscopy was carried out on a Bruker AV600 spectrometer (14.1 T magnetic field strength, operating at 600 MHz for 1H NMR and 125 MHz for 13C NMR spectra).

Water was used from a TKA water purification system (Thermo Scientific, Germany)

Transmission electron microscopy was conducted on a JEM 1010 Microscope (JEOL, Germany) with a 80 kV electron beam or a JEM 2010 Microscope (JEOL, Germany) with a 200 kV electron beam, both using 400 mesh carbon-coated TEM grids cleaned for 45 s with a plasma beam at 25 mA electric current (Elektron Technology, UK, Quorum). Samples were prepared from aqueous solutions at 1 mg/mL. The concentration was obtained by diluting original solutions using water from the TKA water system mentioned above and then stained for 10 s using a 1 M PTA (phosphotungstic acid) solution at pH 7.

2. Experimental Part

2.1. Polymer Synthesis

Briefly, initiator (PEG-Br or ME-Br for M-H-D) and monomer was dissolved in ethanol, the solution degassed for 30 min, and the CuBr/bipyridin mixture added. The polymerisation was left until complete conversion to give a highly viscous mixture. The second block was attached by adding the corresponding amount of ethanol–dissolved monomer to the solution. For M-H-D, this was repeated for the third block. After final conversion, the mixture was filtered over silica gel with ethanol and dialysed against chloroform/methanol (3:1, 2x), methanol (2x), and water (2x) before being freeze-dried. The solvents were 500 mL each time and the time between solvent exchanges was at least 4 h each time.

2.2. GPC traces and NMR spectra

The molecular structure of each copolymer was given in the main paper (Scheme 1). Molecular compositions were calculated taking characteristic peaks of the respective NMR spectra of the polymers and dividing them by the amount of protons they represent. Molecular weights were

calculated from these values and dispersities determined via GPC. Due to either heavy interactions with the column material or solubility issues, PMPC-PHPMA-PDPA could not be analysed via GPC.

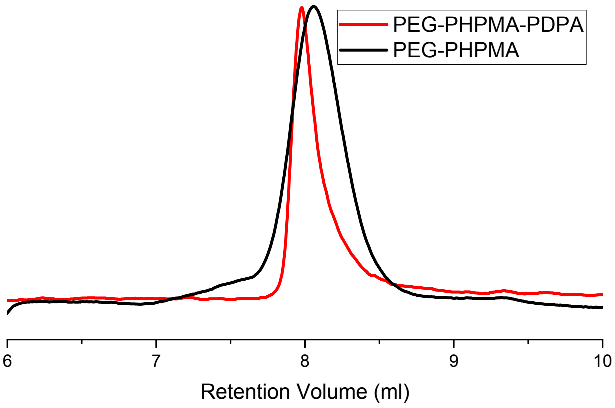

Figure S1. GPC traces for PEG-PHPMA and PEG-PHPMA-PDPA, both recorded in THF.

For PEG_{45}-$PHPMA_{100}$: (H given in number per monomer unit, all broad signals)

^1H NMR (MeOD/CDCl3, 500MHz) δ = 4.74 (1H, HPMA, 3.85 (3H, HPMA), 3.63 (4H, PEG), 1.86 (2H, HPMA), 1.20 (3H, HPMA), 0.89 (3H, HPMA); M_n = 16400 g/mol, Dispersity: 1.24

For PEG_{45}-$PHPMA_{70}$-$PDPA_{30}$: (H given in number per monomer unit, all broad signals)

^1H NMR (MeOD/CDCl3, 500MHz) δ = 4.74 (1H, HPMA), 3.98 (2H, DPA), 3.85 (3H, HPMA), 3.63 (4H, PEG), 2.99 (2H, DPA), 2.64 (2H, DPA), 1.87-1.86 (2H, HPMA, DPA), 1.20 (3H, HPMA), 1.01 (12H, DPA), 0.89 (3H, DPA, HPMA); M_n = 18600 g/mol, Dispersity = 1.15

For $PMPC_{25}$-$PHPMA_{55}$-$PDPA_7$: (H given in number per monomer unit, all broad signals)

^1H NMR (MeOD/CDCl3, 500MHz) δ = 4.74 (1H, HPMA), 4.24 (2H, PMPC), 4.15 (PMPC, 2H) 3.98 (2H, DPA), 3.91 (2H, PMPC), 3.85 (3H, HPMA), 3.70 (2H, PMPC), 3.25 (9H, PMPC) 2.99 (2H, DPA), 2.64 (2H, DPA), 1.87-1.86 (2H, HPMA, DPA), 1.20 (3H, HPMA), 1.01 (12H, DPA), 0.89 (3H, DPA); M_n = 16700g/mol

2.3. Calculating the composition

Reference Peaks taken. Peak assignment as reported previously:

PEG: 3.63 ppm. Intensity was divided by 4 so that the final number represents the intensity of one proton per repeating unit.

PDPA: 2.64 ppm. Intensity was divided by 2 so that the final number represents the intensity of one proton per repeating unit.

PHPMA: 3.85 ppm. Intensity was divided by 2 so that the final number represents the intensity of one proton per repeating unit.

PMPC: 4.24 ppm. Intensity was divided by 2 so that the final number represents the intensity of one proton per repeating unit.

For PEG-based polymers: PEG2000 has 45 repeating units, so the ratio of intensities can be converted to the final number of units present in the polymer. For PMPC, it is assumed that all initiator reacts and as the reaction is run until completion it is assumed that all initiator molecules reacted with 25 units of MPC on average. This number of 25 units is then the basis for all other repeating units in the block-copolymer.

2.4. Typical DLS Traces

In addition to the TEM analysis discussed in the main manuscript, we also checked the DLS traces for the structures, which were relevant. All DLS traces are shown as intensity plots, and the x-axis is the hydrodynamic diameter in nm.

DLS for the micelles observed in Figure 3(b4):

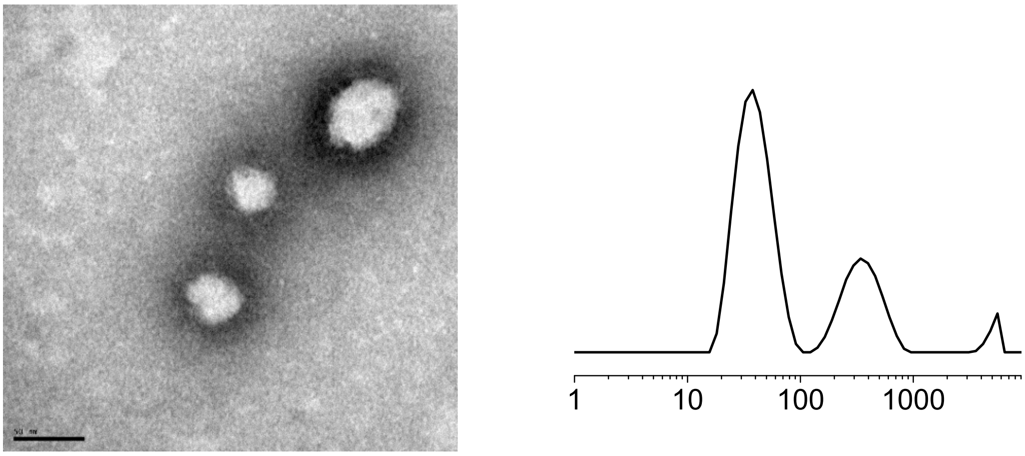

Figure S2. TEM and DLS for the structures observed in Figure 3(b4).

Since larger particles scatter the light more than smaller ones, the DLS appears to show the presence of larger objects as well. However, due to the effect just described, the peak corresponding to agglomerates at 400 nm can be neglected.

Typical DLS trace of the nanobjects observed in Figure 2(b1).

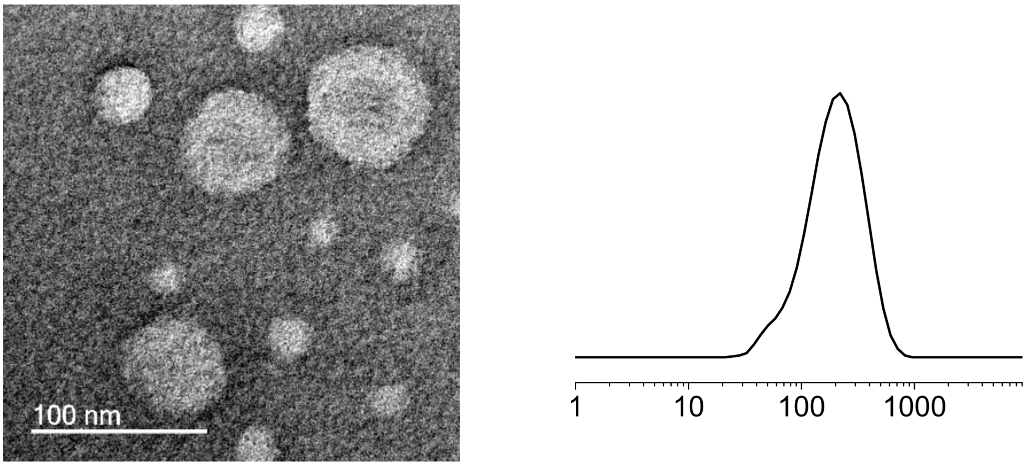

Figure S3. TEM and DLS for the structures observed in Figure 2(b1).

Waffle structures observed in Figure 2(b2):

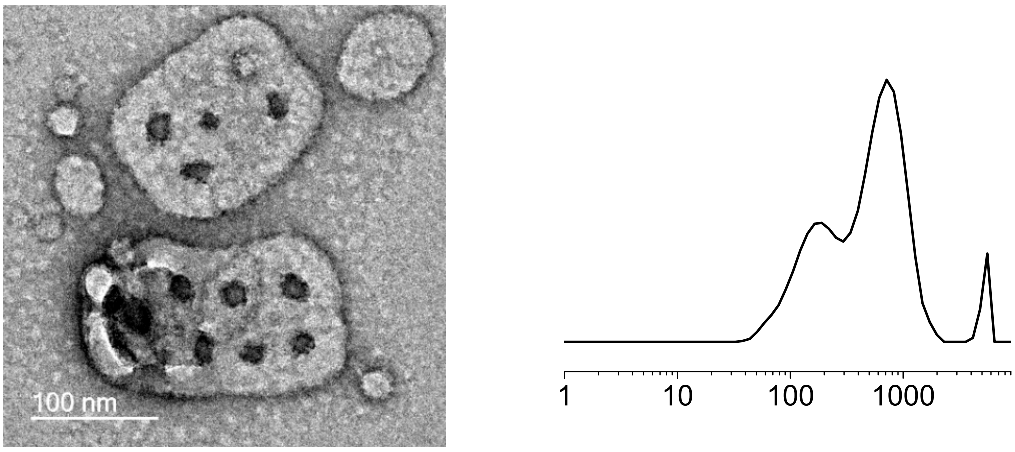

Figure S4. TEM and DLS for the structures observed in Figure 2(b2).

Due to the irregular shape of these nanoparticles (no perfect sphere), the DLS shows two peaks and can be misleading.

Disassembled particles for Figure 2(a4):

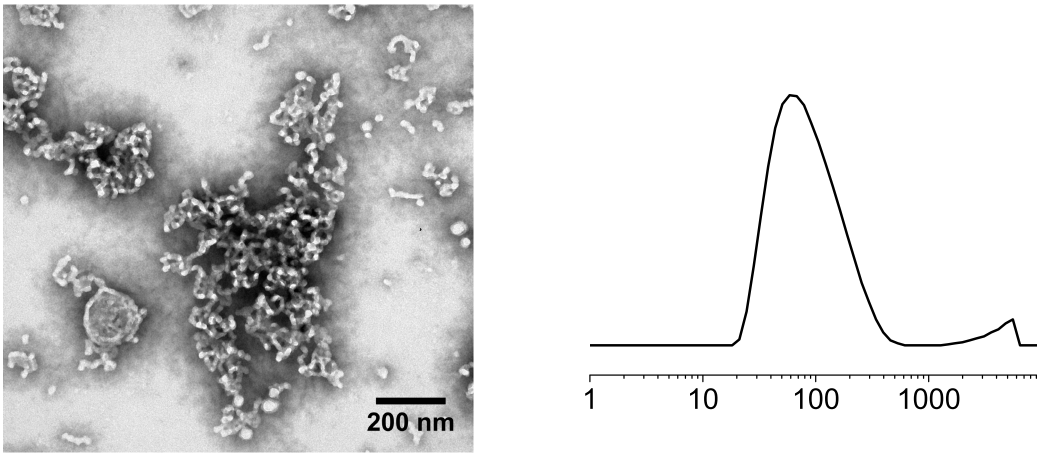

Figure S5. TEM and DLS for the structures observed in Figure 2(a4).

Comparing the image with the DLS trace shows that the DLS trace can be misleading as no defined objects are present.

Half-moon structures observed (for example) in Figure 3(a1):

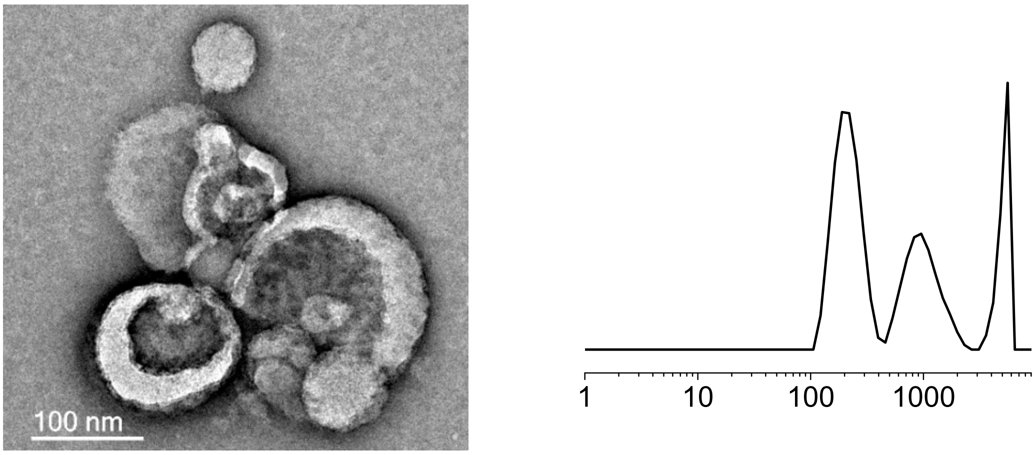

Figure S6. TEM and DLS for the structures observed in Figure 3(a1).

Again, given their irregular shape (no perfect sphere, which would be necessary for DLS), the DLS trace recorded is in itself not very conclusive and may be seen as misleading.

Closed "half-moon" structures of Figure 2(b4):

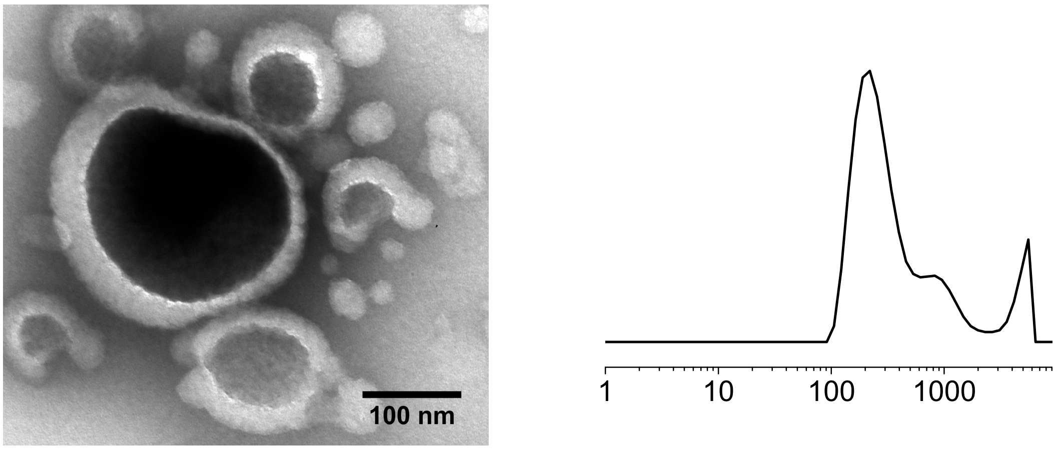

Figure S7. TEM and DLS for the structures observed in Figure 2(b4).

As the structures are now closed, they become spherical again and the DLS corresponds better with the TEM image.

Disassembling half-moon structures of Figure 3(a4):

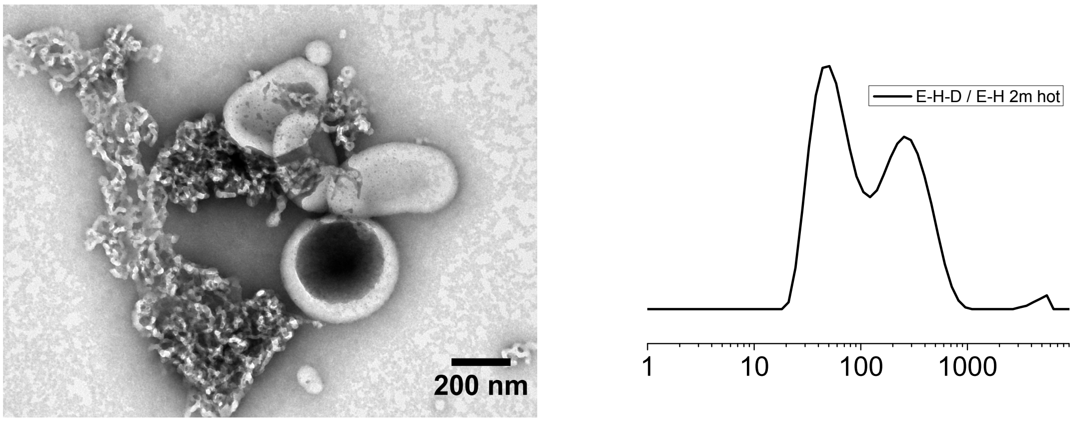

Figure S8. TEM and DLS for the structures observed in Figure 3(a4).

As the structures are disassembling, the distribution becomes even broader and is due to the fact that the present patches of polymer are not conclusive.

2.5. Formation of the Self-Assembly Structures

Ryan et al. published a very nice study on how vesicles are formed using thin film rehydration. This figure represents nicely how the self assembly process works, which is why we included it for reasons of understandability.

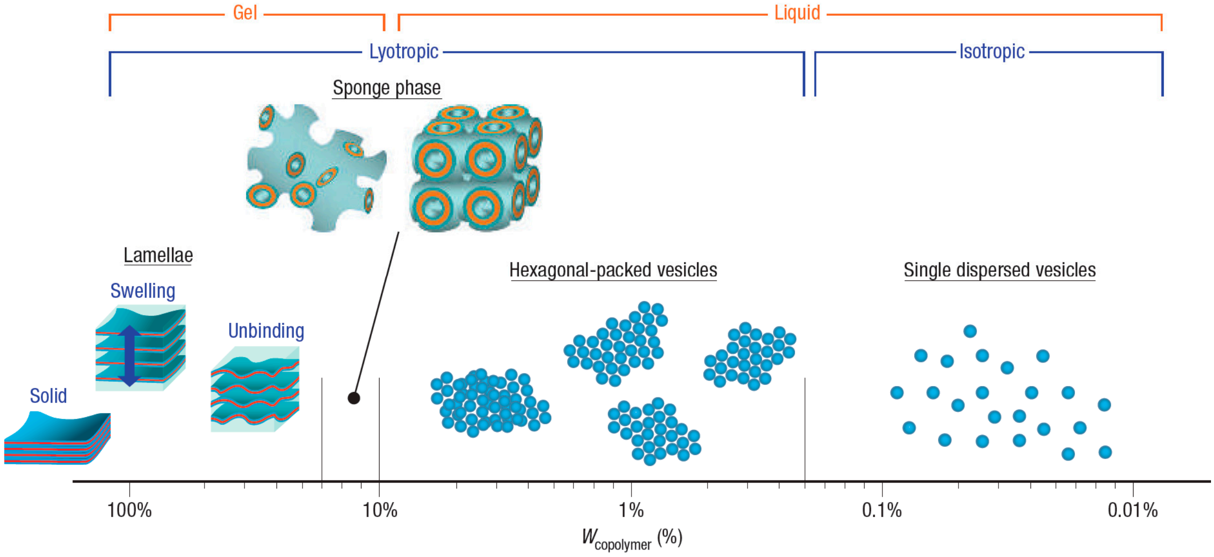

Figure S9. Pathway to vesicles and other self-assembly structures, starting from a thin film. Published with permission from the Nature Publishing Group from [32].
